# Supplementary material for: Hyperbolic optics and superlensing in room-temperature KTN from self-induced k-space topological transitions
Source: Nat Commun. 2021 Dec 13;12:7241. doi: 10.1038/s41467-021-27466-3 (PMC8668897; doi:10.1038/s41467-021-27466-3)
Supplement: Supplementary file 1 — Supplementary Information [file 41467_2021_27466_MOESM1_ESM.pdf]

SUPPLEMENTARY INFORMATION

Hyperbolic optics and superlensing in room-temperature KTN from self-induced k-space topological transitions

Yehonatan Gelkop,[1] Fabrizio Di Mei,[2] Sagi Frishman,[1] Yehudit Garcia,[1,3] Ludovica Falsi,[2,4] Galina Perepelitsa,[1] Claudio Conti,[2,5] Eugenio DelRe,[2,5,*] and Aharon J. Agranat[1,3]

[1] *The Department of Applied Physics, The Hebrew University, Jerusalem 9190401, Israel*

[2] *Dipartimento di Fisica, Università di Roma "La Sapienza", 00185 Rome, Italy*

[3] *The Brojde Center for Innovative Engineering and Computer Science, The Hebrew University, Jerusalem 9190401, Israel*

[4] *Dipartimento S.B.A.I., Sezione di Fisica, Università di Roma "La Sapienza" ,00161 Rome, Italy*

[5] *ISC-CNR, Università di Roma "La Sapienza", 00185 Rome, Italy*

(Dated: November 10, 2021)

* eugenio.delre@uniroma1.it

SUPPLEMENTARY FIGURES

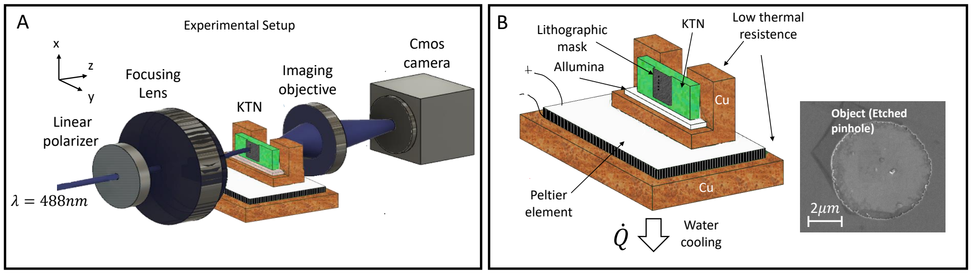

Supplementary Figure 1. (a) Experimental setup and (b) details, including the thermal quenching circuit ($\dot{Q}$ is the heat flux) and the SEM image of the 6 μm diameter 'object' pinhole.

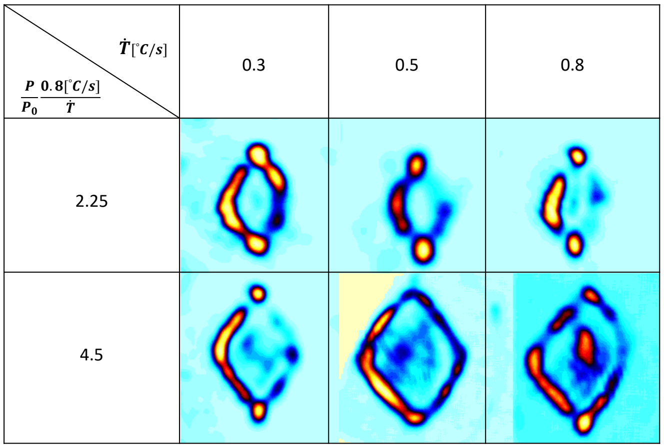

Supplementary Figure 2. Phenomenological analysis of dependence of χ_{PNR} on relative power transmitted at input through the mask pinhole P/P_0 (see main for definition of P_0) and relative cooling rate $\dot{T}/\dot{T}_0$, where $\dot{T}_0 = 0.8°$C/s is the cooling rate used in the main article. Output intensity distribution indicates that an approximately equal value of associated α (not reported here) can be found using different values of $\dot{T}$ using different launch P.

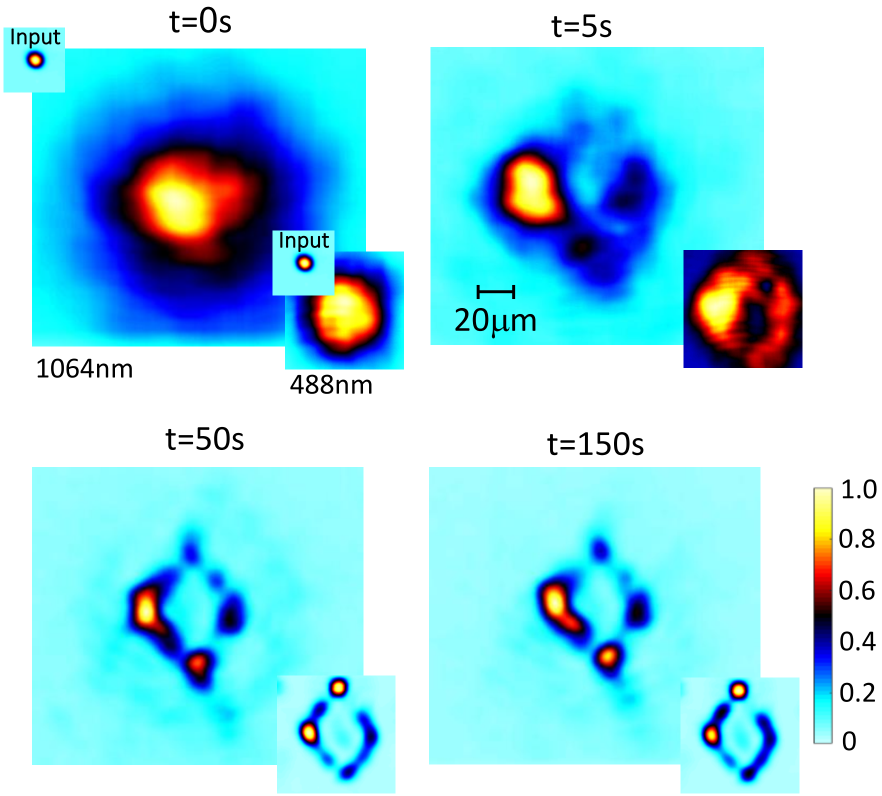

Supplementary Figure 3. Detection of the underlying nonlinear index pattern. (a) Output intensity distribution of an IR beam launched in the sample where a topological transition has occurred at 488 nm for a specific exposure time t and $P = 11\mu\mathrm{W}$ (at 488 nm). (b) Corresponding intensity distribution of the 488 nm beam. The IR linear transmission leads to an enlarged replica of the phenomology observed for the visible beam.

SUPPLEMENTARY DISCUSSION

Hyperbolic dispersion, negative refraction, and superlensing

Hyperbolic dispersion. In a hyperbolic medium, plane wave components $\mathbf{E_k} = \mathbf{E}_0 \exp(i\mathbf{k}\cdot\mathbf{r})$ obey a hyperbolic dispersion relation. Consider, for example, the relation

$$(1-\alpha^2)k_x^2 + (1-\alpha^2)k_y^2 + k_z^2 = k_0^2 n^2. \quad (1)$$

For $\alpha^2 = 0$, Eq.(1) simply represents the standard spherical dispersion manifold of an isotropic dielectric. For values of $0 < \alpha^2 < 1$, waves behave like extraordinary solutions in a positive uniaxial birefringent crystal. The dispersion becomes hyperbolic when $\alpha^2 > 1$, in which case the manifold is a two-sheet hyperboloid, symmetric for rotations around the k_z axis. The corresponding dielectric constant reads

$$\varepsilon_{xx} = \varepsilon_{yy} = \varepsilon_0 n^2, \varepsilon_{zz} = \frac{\varepsilon_0 n^2}{1-\alpha^2}, \varepsilon_{ij} = 0 \text{ for } i \neq j. \quad (2)$$

From Eq.(2), we see that the electric displacement $\mathbf{D} = \overleftrightarrow{\varepsilon} : \mathbf{E}$ is not parallel to the electric field $\mathbf{E}$. Since $\mathbf{k}$ for each plane-wave solution is normal to $\mathbf{D}$ and the associated magnetic field $\mathbf{H}$, while the Poynting vector, $\mathbf{S} = \mathbf{E} \wedge \mathbf{H}$, is normal to $\mathbf{E}$ and $\mathbf{H}$, it follows that $\mathbf{S}$ and $\mathbf{k}$ are not parallel for $\alpha^2 \neq 0$. More to the point, for hyperbolic dispersion, $\varepsilon_{zz} < 0$, and the components of $\mathbf{D}$ and $\mathbf{E}$ along z are flipped one relative to the other. One immediate consequence is that waves with $E_z \neq 0$ whose $\mathbf{k}$ forms, for example, a positive angle with the z axis, have an $\mathbf{S}$ that forms a negative angle with it.

Negative refraction. This basic difference is rendered explicit when waves pass from a material with $\alpha_1^2 = 0$ to one with $\alpha_2^2 > 1$, the boundary between the two being a plane normal to the z axis. In this case, the conservation of the component of $\mathbf{k}_{\perp,1} = \mathbf{k}_{\perp,2}$ parallel to the boundary associated to the continuity conditions on the tangential component of $\mathbf{E}_{t,1} = \mathbf{E}_{t,2}$, paired with the continuity condition of the normal component of $\mathbf{D}_{n,1} = \mathbf{D}_{n,2}$, forces the normal component of the electric field to change both in amplitude and sign

$$E_{z,2} = \frac{\varepsilon_{zz,1}}{\varepsilon_{zz,2}} E_{z,1}. \quad (3)$$

The result is that while $\mathbf{D}$ suffers the standard change in passing from material 1 to material 2 that is associated to the normally observed Snell Law, the electric field flips, relative to

the z axis (parallel to the normal to the boundary), i.e., if $E_{z,1} > 0$ then $E_{z,2} < 0$ (and viceversa). The flip causes $\mathbf{S}$ to bend, relative to the z axis, in a direction opposite to the standard Snell law, leading to what is termed negative refraction.

Constraints on plane-wave description. Reflection and refraction provide a complete picture for conditions in which a single plane-wave-like approximation is sufficient. This is true when the plane-wave spectrum $\mathbf{E_k}$ is such as to explore a region of the dispersion manifold $\mathcal{M}_\mathbf{k}$ of Eq.(1) with a negligible curvature, compared to the actual propagation distances investigated. For a spherical or elliptical $\mathcal{M}_\mathbf{k}$, this leads to the standard diffraction angle $\Delta\theta \sim \lambda/nw_0$, where w_0 is the characteristic width of the wavepacket (or beam), and a plane-wave picture is sufficient typically when $L_z\Delta\theta < w_0$, i.e., the propagation is shorter than the characteristic diffraction length. The picture is modified for a hyperbolic $\mathcal{M}_\mathbf{k}$, since the curvature is finite only for the regions around the axis of the two-sheet hyperboloid (the z axis in the example of Eq.(1)), while the branches of the manifold far from the axis are flat. The actual observation of diffraction effects then requires that the wavepacket spectrum be sufficiently large to observe diffraction, and sufficiently localized so as to populate the parabolic-like region around the hyperboloid axis, i.e., not be dominated by the behavior of the branches.

Wavepackets and group velocity. In conditions then for which the plane-wave-like simplification does not fully describe phenomena and the full diffractive properties of the wavepacket participate in the evolution, the energy flow, identified by the Poynting vector $\mathbf{S}$, follows the dynamics of the wave envelope, i.e., those of the mutual constructive interference pattern generated by the underlying wavepacket components. The interference pattern depends on the relative phase-slippage of the single components, the result being that $\mathbf{S}$ is parallel to wavepacket group velocity, i.e., parallel to the gradient field $\nabla_\mathbf{k}\mathcal{M}_\mathbf{k}$. While for a spherical $\mathcal{M}_\mathbf{k}$ this implies that $\mathbf{S}$ and $\mathbf{k}$ are always parallel and oriented in the same direction, for the two-sheet hyperboloid of Eq.(1), this implies a characteristic negative refraction for the spectrum that populates the hyperbolic branches, and a characteristic antidiffraction for the spectrum that populates the central parabolic region around the z axis.

Superlensing. In the case of a spatial spectrum that is prevalently confined to the parabolic region of the hyperboloid around the z axis, the curvature of the hyperboloid is inverted compared to that of a sphere. Put differently, all the single components are in fact suffering negative refraction relative to the hyperboloid axis, so that the components of the spectrum

that populate, for example, the transverse spectrum with $k_x > 0$, will propagate slanted towards the negative x axis, those with $k_x < 0$, will be slanted towards the positive, and similarly for the k_y components. This constitutes what is generally termed superlensing. The result is that, on exiting the material with $\alpha_2^2 > 1$ back into a material with $\alpha_3^2 = \alpha_1^2 = 0$, each antidiffracted component bends back in a direction compatible with a Poynting vector parallel to $\mathbf{k}$, ultimately causing the wavepacket to acquire a fully unraveled structure after the so-called image, or external focus.

Nonlocality in diffusion-driven response. When light-matter interaction is dominated by photothermal effects or the thermal agitation of photogenerated charges, the nonlinear response becomes spatially nonlocal, depending not only on the optical intensity I, but also on the spatial gradient ∇I. In photorefractive crystals, the space-charge field associated to the thermal diffusion of conduction-band photogenerated electrons reads

$$\mathbf{E}_{dc} = -\frac{k_B T}{q}\frac{\nabla I}{I}, \tag{4}$$

where k_B is the Boltzmann constant, T the temperature, and q the elementary charge. The quadratic electro-optic response is then spatially nonlocal and reads

$$\Delta n = -\frac{1}{2}n^3 g \varepsilon_0^2 \chi_{PNR}^2 |\mathbf{E}_{dc}|^2 = -\frac{1}{2}n^3 g \varepsilon_0^2 \chi_{PNR}^2 \frac{k_B T}{q}\left|\frac{\nabla I}{I}\right|^2. \tag{5}$$

Here g is the effective electro-optic coefficient, and χ_{PNR} is the polar-nano-region-dominated susceptibility.

Shape-dependent nonlinearity. The nonlinear nonlocal response of Eq.(5) scales in a manner that closely mimics diffraction, even though it arises from diffusion. The ratio $|\nabla I/I|^2$ makes it approximately independent of the actual peak beam intensity, and, in turn, proportional to the square of the inverse of the beam width. For a Gaussian-like beam, this behavior is reflected directly in the leading resulting propagation equation

$$((1-\alpha^2)\nabla_\perp^2 + \partial_{zz} + k_0^2 n^2)\mathbf{E} = 0, \tag{6}$$

where $\alpha = L/\lambda$, with $L = 4\pi n^2 \varepsilon_0 \sqrt{g}\chi_{PNR}(k_B T/q)$.

An alteration of $\mathcal{M}_{\mathbf{k}}$. Equation (6) implies that the nonlocal response involves a differential operator, making its interpretation difficult in direct space if compared to local nonlinear effects, such as the Kerr effect, that lead to algebraic expressions in the optical intensity I. For one, the leading wave equation is different from the unperturbed linear equation, but is itself approximately linear. In k-space the effect has an immediate interpretation, as the shape-dependent nonlocal response leads to the hyperbolic dispersion of Eq.(1), with its accompanying negative refraction and superlensing effects.

Passage from one linear wave-equation to another. While the natural interpretation of a diffusion-driven response is in k-space, it can serve a purpose to provide an intuitive

physical interpretation of the model composed of Eqs.(4)-(6) in direct space. Since response is associated to the gradient of the optical intensity, components with ever greater values of transverse $\mathbf{k}_\perp$ suffer an ever stronger phase chirp that ultimately flips the sign of the two transverse spatial terms in the propagation equation (see Eq.(6)). Such a non-perturbative alteration in the governing wave equation is typically encountered in connection with spontaneous-symmetry-breaking. Here, on the basis of a specific control parameter, such as temperature, density, or pressure, strong nonlinear interaction causes a field to become unstable and condense into a once again stable non-interacting state characterized by a different governing linear field equation. The passage is then the basic conceptual framework for the emergence of new particles, mass, charge, and so forth. In the present case, α^2 plays the role of the control parameter. In terms of governing wave equations, the passage occurs from the original space-time wave equation for each field component ϕ

$$\left[-(n/c)^2\partial_{tt} + \nabla_\perp^2 + \partial_{zz}\right]\phi = 0, \tag{7}$$

for $\alpha^2 = 0$ (and $\alpha^2 < 1$), to

$$\left[-(n/c)^2\partial_{t't'} + \nabla_\perp^2 + \partial_{z'z'}\right]\phi = 0, \tag{8}$$

for $\alpha^2 > 1$, with

$$z' = (c/n)(\alpha^2 - 1)^{1/2} t$$
$$t' = (n/c)(\alpha^2 - 1)^{1/2} z.$$

Put differently, the topological transition through the $\alpha^2 = 1$ singularity formally implies the switching of the role of the time axis (t) into a space axis (z'), and the space axis (z), into the time axis (t'). In other words, the passage from a spatial spherical to a hyperbolic manifold can be interpreted as the manifestation of a rotation of the Minkowski manifold.

Cumulative nonlinearity and glass-former response

The photorefractive nonlinearity is the result of the electro-optic response to a photogenerated low frequency space-charge electric field $\mathbf{E}_{sc}$. When no external bias is applied to the sample and photovoltaic effects can be neglected, $\mathbf{E}_{sc}$ is caused by the diffusion of photogenerated mobile carriers from deep donor impurities. Given an optical intensity I, the resulting band-transport model causes $\mathbf{E}_{sc}$ to obey the relationship [1, 2]

$$\tau_d I_b \frac{\partial \mathbf{E}_{sc}}{\partial t} + \mathbf{E}_{sc}(I_b + I) + \frac{k_B T}{q}\nabla(I_b + I) = 0. \tag{9}$$

Here $t_d = \varepsilon\gamma N_a/(q\mu s N_d I_b)$ is the dielectric relaxation time, where ε is the low-frequency dielectric constant (i.e., $\chi_{PNR} = (\varepsilon/\varepsilon_0 - 1)$, γ is the electron-recombination rate, N_a is the sample acceptor impurity density, q is the electron charge, μ is the photoexcited electron mobility in the conduction band, s is associated to the donor absorption cross-section, N_d is the donor impurity density, and I_b is the background (or dark) illumination. The relationship is valid in conditions in which $N \ll N_a \ll N_d$ and charge displacement and saturation terms are neglected. A solution to the this diffusion-driven model is then

$$\mathbf{E}_{sc} = -\frac{\nabla I}{I + I_b}\left(1 - \exp\left(-\int_0^t (1 + I/I_b)dt/t_d\right)\right). \tag{10}$$

The result is that, to a first approximation, the diffusion-driven nonlinearity is governed by

$$\alpha = \frac{L}{\lambda}\left(1 - \exp\left(-\int_0^t (1 + I/I_b)dt/t_d\right)\right). \tag{11}$$

This buildup transient allows us to explore different values of α in time and using different beam peak intensities I_p. For the initial stages of space-charge build-up, that is for $t \ll t_d I_b/I_p$, $\alpha \simeq (L/\lambda)(t_d I_b)^{-1} I_p t$ is proportional to t for a given I_p.

In turn, α can also be controlled, for a given t, by changing the cooling rate $\dot{T}$ and the speed of formation of the space-charge buildup. This is because the sample electro-optic response is mediated by the crystal low-frequency susceptibility that, in quenched KTN, is dominated by the susceptibility of glass-former dynamics of polar nanoregions (PNRs), χ_{PNR}. χ_{PNR} depends on previous thermal history, bias fields during the cooldown, and the actual details of the thermal trajectory, such as the cooling rate $\dot{T}$ and the final equilibration temperature T [3–10]. In the present case, χ_{PNR} is strongly affected by two

factors: i) the cooling rate $\dot{T}$ and ii) the speed of formation of the photogenerated $\mathbf{E}_{sc}$. The latter is, as shown above, approximately proportional to the beam peak intensity (i.e., $\dot{E}_{sc} \propto P$). As reported in Supplementary Figure 2, an approximately constant value of α, as determined by the same output optical intensity distribution, at the same $t = 150$s, is found for approximately constant values of $(P/P_0)(\dot{T}_0/\dot{T})$, where $\dot{T}_0 = 0.8°\mathrm{C}/s$ (the one used in most of the experiments reported in the main article).

Anisotropy

The simplifying assumption at basis of the standard diffusion-driven nonlinear response as described in Ref.[9] is that the PNRs are wholly disordered. In turn, the presence of a thermal gradient will cause a fraction of the PNRs to be aligned. In these conditions we have

$$\chi_{PNR} = \chi_{dis} + \chi_{ord}, \tag{12}$$

where χ_{dis} gives rise to the isotropic response, while χ_{ord} is anisotropic, connected to the PNR alignment and hence direction of the external constraint (the thermal gradient). The optical nonlinearity is associated to the quadratic electro-optic tensor

$$\Delta n_{ij} = -\frac{1}{2} n^3 \varepsilon_0^2 g_{ijkl} \chi_{PNR,k} \chi_{PNR,l} E_{SC,k} E_{SC,l}, \tag{13}$$

assuming that g_{ijkl} is the PNR effective quadratic electro-optic tensor. Taking the g_{ijkl} to coincide with the quadratic tensor of KTN so that only terms with $k = l$ are non-zero (in agreement with the fact that an input linear polarization parallel to the crystal principal axis is not found to change [8, 9]) and neglecting higher-order corrections we have (for the x-polarization)

$$\Delta n_{xx} = \Delta n_{dis} - \frac{1}{2} n^3 \varepsilon_0^2 g_{11} \chi_{ord,x} \chi_{dis} (E_{SC,x})^2 - \frac{1}{2} n^3 \varepsilon_0^2 g_{12} \chi_{ord,y} \chi_{dis} (E_{SC,y})^2. \tag{14}$$

Here Δn_{dis} is the isotropic diffusion-driven nonlinear response [9]. The overall effect is then a different $\alpha_x \neq \alpha_y$, with

$$\alpha_x^2 = 16\pi^2 n^4 \varepsilon_0^2 (K_B T/q)^2 ((g_{11} + g_{12}) \chi_{dis}^2 + g_{11} \chi_{dis} \chi_{ord,x})/\lambda^2, \tag{15}$$

and

$$\alpha_y^2 = 16\pi^2 n^4 \varepsilon_0^2 (K_B T/q)^2 ((g_{11} + g_{12}) \chi_{dis}^2 + g_{12} \chi_{dis} \chi_{ord,y})/\lambda^2. \tag{16}$$

Recalling that $|g_{11}| > |g_{12}|$ and that $g_{11} > 0$ while $g_{12} < 0$, we have that $\alpha_x > \alpha_y$.

IR Readout

A beam splitter is used to combine onto the 488 nm beam (P=11μW, measured as transmitted through the pinhole mask) undergoing the topological transition a second 1064 nm beam (P=20μW). Output transmitted intensity distribution is observed using a low-pass filter that cuts out the 488 nm beam. As in previous experiments, the crystal undergoes rapid cooling exposed to the 488nm laser for different exposure times t, and the effects on the 1064 nm beam are reported in Supplementary Figure 3. If the visible beam is not present, the IR beam diffracts as in a standard homogeneous material, while turning the visible beam off after a given t leaves an imprinted pattern that still affects the IR beam, as $\mathbf{E}_{SC}$ remains imprinted in the material, decaying over long times through dark thermal conductance and residual ambient light.

SUPPLEMENTARY REFERENCES

[1] E. DelRe, P. Di Porto, and B. Crosignani, Photorefractive Solitons and Their Underlying Nonlocal Physics, Progress in Optics 53, 153-200 (2009)

[2] E. DelRe, and M. Segev, Self-Focusing and Solitons in Photorefractive Media, Topics in Applied Physics 114, 547-572 (2009)

[3] Ben Ishai, P., de Oliveira, C. E. M., Ryabov, Y., Feldman, Yu., and Agranat, A. J. Glass-forming liquid kinetics manifested in a KTN: Cu crystal. Phys. Rev. B 70, 132104 (2004).

[4] Bitton, G., Razvag, M., and Agranat, A. J. Formation of metastable ferroelectric clusters in $K_{1-x}Li_xTa_{1-y}Nb_yO_3$:Cu,V at the paraelectric phase. Phys. Rev. B 58, 5282-5286 (1998).

[5] Chang, Y.-C., Wang, C., Yin, S., Hoffman, R. C., and Mott, A. G. Giant electro-optic effect in nanodisordered KTN crystals. Opt. Lett. 38, 4574-4577 (2013).

[6] Chang, Y-C., Wang, C., Yin, S., Hoffman, R. C., and Mott, A. G. Kovacs effect enhanced broadband large field of view electro-optic modulators in nanodisordered KTN crystals. Opt. Express 21, 17760-17768 (2013).

[7] Pierangeli, D., Parravicini, J., DiMei, F., Parravicini, GB., Agranat, A. J., and DelRe, E. Photorefractive light needles in glassy nanodisordered KNTN. Opt. Lett. 39, 1657-1660 (2014).

[8] Gumennik, A., Kurzweil-Segev, Y., and Agranat, A. J. Electrooptical effects in glass forming liquids of dipolar nano-clusters embedded in a paraelectric environment. Opt. Mat. Expr. 1, 803-815 (2011).

[9] DelRe, E., Spinozzi, E., Agranat, A. J., and Conti, C. Scale-free optics and diffractionless waves in nanodisordered ferroelectrics. Nat. Photon. 5, 39-42 (2011).

[10] DelRe, E., Di Mei, F., Parravicini, J., Parravicini, GB., Agranat, A.J., and Conti, C. Sub-wavelength anti-diffracting beams propagating over more than 1,000 Rayleigh lengths. Nat. Photon. 9, 228-232 (2015).
