# Supplementary material for: Hyperbolic optics and superlensing in room-temperature KTN from self-induced k-space topological transitions
Source: Nat Commun. 2021 Dec 13;12:7241. doi: 10.1038/s41467-021-27466-3 (PMC8668897; doi:10.1038/s41467-021-27466-3)
Supplement: Supplementary file 3 — Description of Additional Supplementary Files [file 41467_2021_27466_MOESM3_ESM.pdf]

File name: Supplementary Data 1

Description: Dataset for graph in Figure 2 panel E
